# A close‐up view of dynamic biomarkers in the setting of COVID‐19: Striking focus on cardiovascular system

**DOI:** 10.1111/jcmm.17122

**Published:** 2021-12-11

**Authors:** Aysa Rezabakhsh, Seyyed‐Reza Sadat‐Ebrahimi, Alireza Ala, Seyed Mohammad Nabavi, Maciej Banach, Samad Ghaffari

**Affiliations:** ^1^ Cardiovascular Research Center Tabriz University of Medical Sciences Tabriz Iran; ^2^ Emergency Medicine Research Team Tabriz University of Medical Sciences Tabriz Iran; ^3^ Applied Biotechnology Research Center Baqiyatallah University of Medical Sciences Tehran Iran; ^4^ Department of Hypertension, Chair of Nephrology and Hypertension Medical University of Lodz Lodz Poland; ^5^ Polish Mother’s Memorial Hospital Research Institute (PMMHRI) Lodz Poland

**Keywords:** cardiac biomarkers, cardiovascular system, COVID‐19, laboratory biomarkers, SARS‐CoV‐2

## Abstract

Based on the recent reports, cardiovascular events encompass a large portion of the mortality caused by the COVID‐19 pandemic, which drawn cardiologists into the management of the admitted ill patients. Given that common laboratory values may provide key insights into the illness caused by the life‐threatening SARS‐CoV‐2 virus, it would be more helpful for screening, clinical management and on‐time therapeutic strategies. Commensurate with these issues, this review article aimed to discuss the dynamic changes of the common laboratory parameters during COVID‐19 and their association with cardiovascular diseases. Besides, the values that changed in the early stage of the disease were considered and monitored during the recovery process. The time required for returning biomarkers to basal levels was also discussed. Finally, of particular interest, we tended to abridge the latest updates regarding the cardiovascular biomarkers as prognostic and diagnostic criteria to determine the severity of COVID‐19.

## 1 INTRODUCTION

**FIGURE 1:**
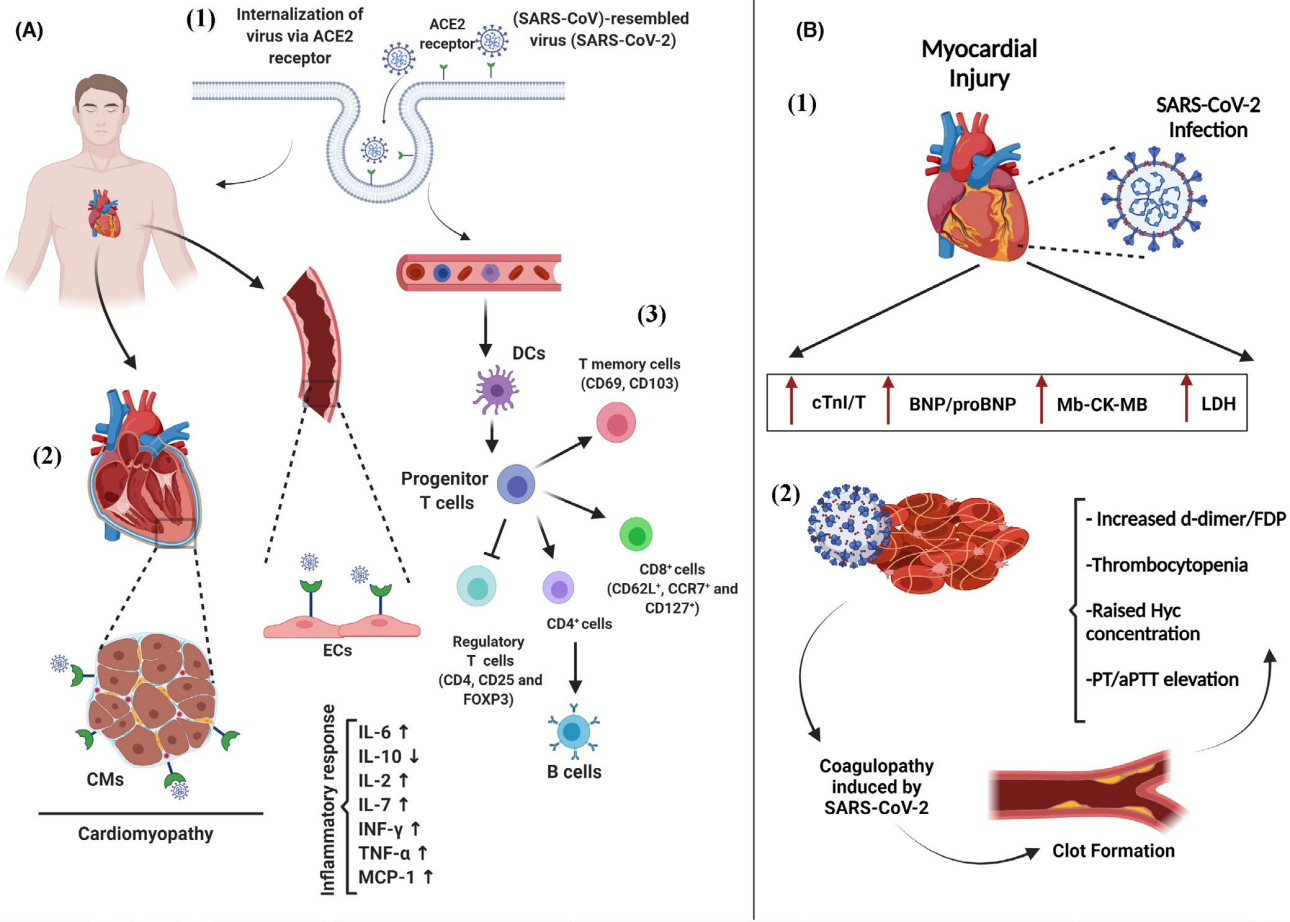
Mechanism of action of coronavirus. 1. The rout of virus transmission and cellular infection via binding to the angiotensin‐converting enzyme 2 (ACE2) receptors expressed ubiquitously in pneumocyte, cardiomyocytes and endothelial cells, 2. CV injury induced by SARS‐CoV‐2 virus, 3. At late phase and severe form of the disease, hyperactivity of inflammatory responses could be observed which characterize by cytokine storms. Coronavirus by targeting the dendritic cells (DC) suppresses the various T‐cell subsets and propagates inflammatory responses by cytokines release such as interleukin‐6 (IL‐6), IL‐10, IL‐2 and IL‐7 as well as interferon‐gamma (INF‐γ), tumour necrosis factor‐alpha (TNF‐α) and monocyte chemoattractant protein‐1 (MCP‐1) (A). The effect of SARS‐CoV‐2 virus on the CV system and concomitantly leads to cardiomyopathy that is detectable through specific cardiac biomarkers measurement. 1. All specific cardiac biomarkers including cardiac troponin I/T (cTn I/T), lactate dehydrogenase (LDH) enzyme, myoglobin (Mb), creatine kinase‐MB (CK‐MB) and brain natriuretic peptide (BNP)/NT‐proBNP represent elevated levels following the manifestations of the clinical symptoms. 2. Following COVID‐19 infection, coagulation events frequently occur particularly in non‐survivors, and clot formation increases due to the coagulopathy determined by elevated levels of d‐dimer/FDP, PTT/aPTT, Hyc and fibrinogen (B). The figure was created with BioRender.com

Moment‐by‐moment reports exhibit the pandemic outbreak of new emerging coronavirus disease 2019 (COVID‐19) caused by severe acute respiratory syndrome coronavirus 2 (SARS‐CoV‐2), which leads to over 4.55 million deaths worldwide.^1^ There is no doubt that different cell types, particularly type II pneumocytes, with the ability to express transmembrane receptors, so‐called angiotensin‐converting enzyme 2 (ACE2) and transmembrane protease serine 2 (TMPRSS2), are at the centre of a SARS‐COV‐2 attack (Figure 1A).^2^, ^3^ Considering the relatively high expression levels of ACE2 and TMPRSS2 in cardiomyocytes, it should come as no surprise that a significant proportion of the COVID‐19 mortality is associated with cardiovascular disease (CVD), in which morbidity and mortality rates can increase in individuals with a history of cardiovascular (CV) co‐morbidities.^4^ In this sense, Razeghian‐Jahromi et al. also emphasized the importance of the dichotomous role of ACE2 regulation on the COVID‐19 progression and subsequent SARS‐CoV‐2‐induced CV complications, particularly in positive patients with heart failure (HF).^5^

Of note, in patients with early infection of the lung tissue, the expansion of COVID‐19 in the CV system could increase the risk of death (Figure 1A).^6^ Calling attention, the coincident occurrence of sepsis, hypoxia and haemodynamic failure predisposes the COVID‐19 patients to myocardial injuries, as well as cardiac arrhythmias.^7^ Besides, patients who receive medications for various reasons (eg QT‐prolonging drugs) are likely to be at higher risk of cardiac arrhythmia upon the COVID‐19.^8^ In this regard, the exploration of the presumable link between various underlying causalities and CVD (such as myocardial infarction (MI) and cardiac arrhythmias) is a crucial issue at the time of COVID‐19 to decline the mortality statistics.^9^ A prospective cohort study performed in Wuhan pulmonary hospital introduced the major risk factors involved in SARS‐COV2 mortality, including age 65 years and over, pre‐existing CV or cerebrovascular diseases comorbidity, the low count of CD3^+^/CD8^+^ T cells (≤75 cell/μl) and elevation of cardiac troponin I (cTnI) (≥0.05 ng/μl).^10^ Nowadays, identifying the people at risk for the severe form of COVID‐19 is one of the concerning issues, considering a global health priority. Therefore, the current review article is a preliminary attempt to address the early prediction of cardiac injury by measuring specific biomarkers following the disease diagnosis, which enables clinicians to raise therapeutic efficiency in vulnerable patients. Herein, we sought to focus on haematological, inflammatory and CV‐related dynamic biomarkers following the COVID‐19 infection:

## 2 COVID‐19‐RELATED PARA‐CLINICAL PROGNOSTIC AND DIAGNOSTIC VALUES

Given that COVID‐19 infection leads to the imbalance in both haematopoietic and haemostasis systems, thereby, laboratory biomarkers, including haematological, biochemical and inflammation parameters, would be more effective criteria for initial screening of the COVID‐19 positive patients^11^:

### 2.1 Haematological biomarkers

As preliminary measures, the assessment of haematological parameters is considered the key indicators for the early diagnosis of critically ill patients. The commonly used haematological patterns, consisting of white cell count (WCC), lymphocyte (Lymph), neutrophil (N), neutrophil‐lymphocyte ratio (NLR), eosinophil, basophil, platelet (Plt) count and haemoglobin (Hb), are assessed in the COVID‐19 patients.^12^ At the early stage of the disease, the results of the complete blood count (CBC) test indicate lymphopenia as a reliable indicator that varies between 25% in mild and 80% in severe forms.^13^ Li et al. also claimed that serum amyloid A (SAA) is considered a sensitive indicator along with lymphopenia in evaluating the severity and prognosis of COVID‐19 patients. Notably, monitoring the SAA dynamic changes along with chest computed tomography (CT) imaging could be a valuable criterion in diagnosing and treating COVID‐19.^14^ In addition, the low count of plt (thrombocytopenia) underlined the poor prognosis of the disease during hospitalization and has been found one of the leading causes of raised mortality besides the acute respiratory distress syndrome (ARDS).^15^ In this line, a systematic review also demonstrated that thrombocytopenia is most likely observed in non‐survivor than the survivor patients.^16^

In contrast, the results of a clinical study on 553 hospitalized SARS‐CoV‐2 positive patients showed that the plt count was significantly higher than in those with severe pneumonia induced by non‐SARS‐COV‐2 causalities (215 ± 100 vs. 188 ± 98, ×109/L, *p* = 0.015).^17^ One of the reasons for plt count changing during COVID‐19 is probably related to the damaged pulmonary endothelial cells, which play a major role in triggering the excessive activation of the lung plt count and subsequently developing the micro‐thrombi formation.^18^ NLR is frequently calculated to predict CVD and sepsis‐related mortality rate and counts as an independent indicator for detecting the severe form of COVID‐19.^19^ Consequently, it has been presumed that the dynamic changes in the haematological parameters may directly attack the bone marrow precursors and hence could promote an auto‐immune response against the blood cells by affecting the haematopoiesis.

Recently, monocyte distribution width (MDW) is also defined as a novel indicator for sepsis or other infection diagnoses, which is remarkably increased during the complicated COVID‐19 infection.^20^ Also, red blood cell distribution width (RDW), a well‐known value for predicting clinical outcomes in many disorders such as trauma,^21^ stroke and pulmonary diseases,^22^ has been validated as a reliable prognostic biomarker for monitoring the severity of ill patients.^23^ In this regard, the data extracted from a meta‐analysis revealed that the mean of absolute RDW value in critical form was 0.69% higher than the patients with a mild form of COVID‐19 (95% CI, 0.40–0.98%; *p* < 0.001).^24^ Additionally, patients with increased RDW (>14.5%) had an approximately threefold increase in the risk of mortality during hospitalization when compared to the patients with normal RDW.^25^ There is also some evidence for the association of elevated baseline RDW with therapeutic failure in response to thrombolytic therapy in patients with ST‐elevated myocardial infarction (STEMI), suggesting an impaired microcirculatory function as a target in both conditions.^26^

### 2.2 Biochemical and inflammatory biomarkers

In the case of the biochemical parameters, C‐reactive protein (CRP) is one of the major biomarkers reflecting inflammatory responses at the acute phase of the disease. Moreover, CRP is positively associated with the increased severity rate upon all infectious diseases due to the uncontrolled inflammation responses.^27^ Meanwhile, it could be a more reliable predictor with high sensitivity and specificity in the earlier stage of the disease, even before chest CT scan findings.^28^ Notably, it has been suggested that the lymph (number/μl)/CRP (mg/dl) ratio is also touted as another valuable predictor showing the excessive inflammatory responses, which significantly decreases in the severe form of COVID‐19.^29^ Also, the results of a recent meta‐analysis performed on 4,663 patients further highlighted that patients with elevated levels of CRP and/or lactate dehydrogenase (LDH) need more intensive care.^30^

Besides the decreased level of both eosinophils and basophils, the study of 450 positive patients reported that a significantly lower rate of different subsets of T cells and B cells is also considered as poor prognostic findings, especially in the severe and fatal forms of the COVID‐19.^31^ Similarly, the results of a clinical study also showed the dynamic changes of the various T cells subsets, including CD3^+^, CD4^+^, CD8^+^, CD19^+^ and CD16^+^/56^+^ lineages, which were lower than the normal reference range 7 days after the onset of disease symptoms, reached the nadir within the 17 days (Figure 1A).^32^ In addition, the results revealed that the ratio of CD3^+^/CD4^+^ and CD3^+^/CD8^+^ was lower in both non‐survivor patients and severe forms of the disease, highlighting the necessity of T‐cell‐based immunotherapy.^32^ Among the inflammatory cytokines produced during the illness, interleukin‐6 (IL‐6) is an abundant cytokine that robustly rises (~threefold) during the cytokine storm in complicated COVID‐19.^33^ Furthermore, other cytokines such as IL‐2, IL‐7, tumour necrosis factor‐alpha (TNF‐α), interferon (IFN)‐γ, macrophage inflammatory protein (MIP)‐1α, inducible protein (IP)‐10, monocyte chemoattractant protein (MCP)‐1 and granulocyte‐colony stimulating factor (G‐CSF) also increased in the course of COVID‐19.^34^

### 2.3 Discriminative values for bacterial infection

A pro‐peptide of calcitonin named pro‐calcitonin (PCT) that has a high similarity with other cytokines such as IL‐6 and TNF‐alpha family is not detectible in the sera of healthy people (<0.1 ng/ml).^35^ However, PCT could rise to 10‐fold (to over 100 ng/ml) due to the extra‐thyroid organ production following the various bacterial infections or tissue damage such as the liver, pancreas, kidney, lung, intestine and leukocytes.^36^ The increased level of PCT presumably reflects the bacterial co‐infection in patients with a severe form of COVID‐19. In this line, a study conducted by Italian researchers reported that following the bacterial infection and elevated PCT levels, the risk of severe COVID‐19 progression was estimated to be about five times higher.^37^

## 3 IMMINENT CV EVENTS FOLLOWING COVID‐19 INFECTION

To our knowledge, the evolving data have emphasized that in patients with CVD comorbidity, the possibility of the COVID‐19 occurrence is more, classifying it as one of the independent risk factors of mortality besides the ARDS, particularly in elderly patients (mortality rate in CVD vs. non‐CVD patients =51% vs. 5%).^38^ In this regard, myocardial injuries could occur through a variety of mechanisms, including a) direct transmission of the virus into the cardiomyocytes (Figure 1B), b) pneumonia‐induced severe hypoxia triggering cardiac ischaemia and c) the overwhelming inflammatory responses (cytokine storm).^39^ Here, we outlined the concerning cardiac biomarkers altered during the COVID‐19 infection:

### 3.1 Effects of COVID‐19 on cardiac biomarkers

#### 3.1.1 Troponins (Tns)

Troponins consist of three regulatory and functional proteins, including Tn‐C, Tn‐I and Tn‐T, which promote contraction in skeletal and cardiac muscles. Regarding the cTn, various conditions such as viral myocarditis, microangiopathy, renin‐angiotensin system (RAS) impairment, hypoxia, inflammatory storm and cytokine‐driven myocardial damage are participated to drive serum cTn elevation in the course of the COVID‐19 infection.^40^

##### Cardiac troponin I (cTnI)

Recently, there has been great interest in using the high sensitivity c‐troponin I (hs‐cTnI) to diagnose and prognosis coronary artery disease (CAD), HF and other CV‐related abnormalities.^41^ Also, it is appeared to be a preferable biomarker to predict the upcoming CV events in the general population.^42^ Given that the elevated cTn concentrations represent the cardiac injury, a meta‐analysis was performed to determine cTnI capability in predicting the COVID‐19 severity. The results showed that despite the high heterogeneity among included studies (I^2^ = 98%; *p* < 0.001), the levels of cTnI value were found to be significantly enhanced in the severe vs. the non‐severe COVID‐19 patients.^43^ Another meta‐analysis also revealed that in non‐survivors, high levels of cTnI were detected (weighted mean difference [WMD] =32.7 ng/L).^31^ Accordingly, the analysed data of 2,736 patients demonstrated that there was a linear correlation with the cTnI >0.09 ng/dl concentration and higher pre‐existing CVD, illness severity, poor prognosis and the highest mortality rate (adjusted hazard ratio (HR) 3.03, 95% CI 2.42–3.80; *p* < 0.001).^44^ In this respect, some evidence suggested that a significantly high rate of cTnI induced by cardiac dysfunction could be considered an independent predictor of COVID‐19 mortality.

In a multi‐centre retrospective cohort study, the levels of median hs‐cTnI were ~8.8 pg/ml in non‐survivor vs. ~2.5 pg/ml in survivor patients 4 days upon the onset of the symptoms. It is worth noting that the amount of hs‐cTnI did not change significantly among the survivors, while it sharply rose to 290.6 pg/ml in non‐survivors after 22 days.^45^ Besides, the study of 416 hospitalized patients reported that hs‐cTnI was raised in 1 of 5 patients during hospitalization, who were more likely to require invasive or non‐invasive ventilation, ARDS or acute kidney injury.^46^ Therefore, it could be reasonable to propose that the measurement of cardiac biomarkers immediately after the hospitalization for SARS‐CoV‐2 infection, as well as inpatients longitudinal monitoring, may be helpful to identify a proportion of patients with possible cardiac damage and thereby predict the progression of COVID‐19 towards a poor prognosis, triage to a critical care area, and indicates the need for inotropes and vasopressors application. On the other hand, patients with either long‐term CAD or atherosclerotic CVD have an augmented risk of developing an acute coronary syndrome (ACS) during acute infections due to the increase in myocardial demand,^39^ which has been shown previously in viral infections induced by influenza virus.^47^, ^48^, ^49^

Underlying mechanisms involved in ACS development in respiratory infections such as pro‐inflammatory and pro‐thrombotic states are well‐described in a comprehensive review performed by Schiavone and his colleagues.^50^ Worse to mention, the increased level of cTnI derived from the viral myocarditis and/or secondary myocardial injury following the COVID‐19 infection has imposed coronary syndrome in these patients. Likewise, in a case report describing an admitted COVID‐19 patient in ICU, ARDS was observed with inverted T waves in the ECG along with a sharply elevated hs‐cTnI concentration (900.2 ng/dl).^51^ In this regard, the clinicians inevitably decided first to roll out the diagnosis of non‐STEMI using coronary angiography and then perform CV magnetic resonance scanning, which detected sub‐epicardial late gadolinium enhancement of the apex and inferolateral wall (suggestive of myocarditis).^51^ Therefore, the authors suggested it is crucial to consider coronary angiography in certain subjects in those with high Global Registry of Acute Coronary Events (GRACE) risk score or with dynamic ECG changes.^51^ Another study conducted by Huang et al. also showed that approximately 12% of patients with SARS‐CoV‐2 were diagnosed as having an acute myocardial injury, mainly manifested by elevated levels of hs‐cTnI.^52^ Of note, there is a lack of compelling evidence for managing patients with dyspnoea with a probable diagnosis of non‐STEMI and COVID‐19. Therefore, the applied guidelines are warranted to be adopted, specifically for managing of similar patients.^47^, ^48^, ^49^

##### Cardiac troponin T (cTnT)

In addition to cTnI, some case series also reported an elevated hs‐cTnT with other functional and biochemical changes in the heart tissue. According to the results of a recent study, the CV complications and elevated levels of cTnT value have been observed in 35.3% and 27.8% of positive patients respectively.^52^ Moreover, increased cTnT has been demonstrated to be linearly correlated with stimulated inflammatory markers indicating that myocardial injury is most likely related to the underlying inflammatory responses.^53^ More importantly, the mortality rate was remarkably higher in patients with elevated cTnT levels compared to patients with lower cTnT during the hospital stay. In line with this, a prospective cohort study also reported that among 177 patients, 119 cases (67%) showed elevated cTnT levels (> 0.03 ng/ml), which were significantly associated with the mortality rate (*p* = 0.008). To further substantiate the relationship between cTnT and mortality rate, authors unveiled that there was a significant correlation between the elevated cTnT level and increased number of organ failures, echocardiographic wall motion abnormalities and the severity of tricuspid regurgitation (adjusted HR =1.45, 95% CI 1.17–1.81, *p* = 0.001).^54^ It has also been reported that the level of cTnT value frequently elevates among the patients with ARDS and in older individuals in those with predisposing conditions, for example hypertension, diabetes, cardiomyopathy, coronary heart disease and chronic kidney disease.^55^ Accordingly, an increased level of cTnT reflects poor clinical outcomes accompanied by echocardiographic abnormalities.^55^ These findings further highlight that a positive history of CVD can precipitate poor clinical outcomes, and secondary cardiac injury derived by COVID‐19 infection is also directly associated with the higher mortality rate.^53^, ^55^ Therefore, it can be applied for risk stratification at admission and disease progression assessment in hospitalized patients during their hospital stay.

The diagnostic challenges regarding the differentiation between ACS and myocarditis/secondary MI observed in COVID‐19 patients with elevated cTnI still exist. In this line, a case report also explained a COVID‐19 positive patient referred with chest discomfort and shortness of breath, significant elevation of cTnT, ST‐segment elevation in the leads aVF and III, and acute HF (LVEF <27%). However, an emergency coronary CT angiography revealed no coronary stenosis, and a final diagnosis of myocarditis was given.^56^ Consequently, existing guidelines in terms of the ACS should consider the possible effects of COVID‐19 on the level of cTnT.

#### 3.1.2 Brain natriuretic peptide (BNP)/N‐terminal‐proBNP (NT‐proBNP)

Brain natriuretic peptide and its inactive pro‐hormone, NT‐proBNP, are typically produced by cardiomyocytes in the baseline range of <125 pg/ml in patients with 0–74 years and <450 pg/ml in elderly subjects aged 75–99 years.^57^ The extent of ventricular haemodynamic stress signifies the alternation in cardiac pressure, which subsequently induces the excessive BNP release and spontaneous myocardial stress.^58^ Regarding the COVID‐19, recent clinical studies reported that the level of NT‐pro‐BNP supposedly increased in patients admitted to ICU.^59^ The study of Gao et al. also reported a sensitivity of 100% and specificity of 66.67% for high NT‐proBNP value consideration (>88.64 pg/ml) in predicting mortality rate in COVID‐19 patients.^60^ The dynamic changes of NT‐proBNP during hospitalization are also an indicator of poor clinical outcome. Moreover, COVID‐19 patients who expired during hospitalization had an increased level of NT‐proBNP during their hospital stay compared to admission.^53^

#### 3.1.3 Dysregulation of heart‐brain axis in COVID‐19

In critically ill patients, developing heart‐brain axis (HBA) dysfunction during SARS‐CoV‐2 infection through neurotropism and psychosocial factors alternation along with cerebral small vessel disease is also regarded as a great concern, which can ultimately lead to CVD. It has been deciphered that HBA dysfunction also can worsen the COVID‐19 complications and contributes to multi‐organ disease syndrome, particularly in patients with ARDS who were admitted in ICU or even after hospital discharge.^61^ Microglia stimulation can exert following the IL‐1β and IL‐6 increased levels, blood‐brain barrier disruption and endothelial dysfunction upon the cytokine storm. On the other hand, increased levels of Toll‐like receptors (TLRs) and damage‐associated proteins derived from dying neurons can exacerbate inflammatory responses induced by cytokine storm and subsequently lead to CV complications (eg cardiac inflammation and HF). Nevertheless, considering the HBA protection would be a more helpful target for personalized therapeutic approaches.^61^

#### 3.1.4 Myoglobin (Mb) and creatine kinase myocardial band (CK‐MB)

For early diagnosis of possible acute MI in patients with COVID‐19, after the onset of the symptoms, Mb and CK‐MB in time intervals 2–6 and 12–24 h, respectively, are well‐suited values with more sensitivity.^62^ The results of recent meta‐analysis revealed that the increased levels of CK‐MB (WMD =2.60 U/L, 95% CI =1.32–3.88, *p* < 0.001) and Mb (WMD =159.77 ng/ml, 95% CI =99.54–220.01, *p* < 0.001) were considerably associated with a significant increase in the severity and mortality of COVID‐19 infection respectively.^63^ Moreover, a multi‐centred retrospective study with 3,219 admitted patients evaluated the predictive power of some cardiac biomarkers in COVID‐19 using the mixed‐effects Cox model. The results of this study showed that the adjusted HR of 28‐day mortality for Mb and CK‐MB was 4.86 ([95% CI, 3.33–7.09] *p* < 0.001) and 4.50 ([95% CI, 3.18–6.36], *p* < 0.001), respectively, which implicated the possible relationship between these two parameters and disease progression.^59^ Besides, the authors indicated that the cut‐off for cardiac values in the case of the 28‐day mortality was found to be 19–50% lower than established thresholds for other regular heart diseases.^59^ Similarly, a systematic review declared that elevated levels of CK‐MB were observed among 12% of patients with COVID‐19.^64^ In parallel with these findings, the results of a meta‐analysis in deceased patients also confirmed the significant high levels of Mb (SMD =1.64, 95% CI 1.34–1.93, *p* < 0.001).^65^

### 3.2 Effects of COVID‐19 on coagulation biomarkers

#### 3.2.1 D‐dimer

D‐dimer is a small fragment protein that originates from the decomposed cross‐linked fibrins during the coagulopathy processes. The elevated levels of d‐dimer generally indicate coagulation and fibrinolysis over‐activity under the multitude of pathological conditions such as disseminated intravascular coagulopathy (DIC), pulmonary embolism, deep venous thrombosis (DVT) and sepsis.^66^ Calling attention, the increased inflammatory markers (eg CRP and IL‐6) are thought to be substantial risk factors in developing systemic vasculitis, leading to defects in the coagulation processes and finally resulting in parenchymal lesions in vital organs such as the CV system.^67^ Among the haemostatic abnormalities in COVID‐19, d‐dimer should be measured as a coagulopathy index. According to the compelling evidence, up to 90% of hospitalized patients with ARDS were concurrent with the coagulation over‐activity in a wide array of vascular beds and subsequent elevated d‐dimer level.^68^ Notably, the results of a recent multi‐centre retrospective cohort study showed that the higher levels of d‐dimer >1 g/L were directly associated with the poor prognosis and inpatients mortality rate. Also, 71.4% of ill patients were faced with a critical condition due to the DIC phenomenon.^69^ To improve outcomes in these cases, the paucity of data declared that optimal thrombo‐prophylactic regimen such as prophylactic doses of anticoagulant medications (unfractionated or low molecular weight heparin) could contribute to reducing the mortality rate.^70^

#### 3.2.2 Platelets

It has been presumed that one of the main factors in the progression of the CV manifestations following COVID‐19 infection refers to the morphological changes in the plt count. In this context, some intracellular modifications were also reported; for instance, the *P*‐*selectin* gene was up‐regulated under the COVID‐19 infection, which could trigger the subsequent heart attack and stroke occurrence following the exacerbated plt aggregation.^71^ It has also been proposed that plt hyperactivity stimulates the mitogen‐activated protein kinase‐related signalling pathway and thromboxane generation. To discriminate from the typical acute DIC, both plt count and fibrinogen alternation should be considered in the COVID‐19‐related coagulopathy with vaso‐occlusive clots or chilblain‐like presentation. Noteworthy, the increased inflammatory and pro‐coagulant responses following COVID‐19 infection enhance the risk of non‐ischaemic MI along with the secondary MI due to the respiratory failure‐induced hypoxia and haemodynamic instability in hospitalized patients with poor prognosis.^72^ Moreover, some critical factors such as prolonged prothrombin time (PPT), higher international normalized ratio (INR) and thrombin time (TT) indicate coagulopathy enhancement over the course of disease.^73^ Moreover, in the initial stage of coagulopathy, the evaluation of d‐dimer and fibrin/fibrinogen degradation products but not PTT and plt count would be more useful para‐clinical indicators. In Table 1, divergent parameters have been listed according to the risk stratification. Moreover, in Figure 1B and Table 2, cardiac and coagulation biomarkers modification following the COVID‐19 progression have been illustrated.

**TABLE 1:** Risk stratification of the biomarkers according to required priority

Indicators	Acute phase	Prognostic of high risk patients	Specific for cardiac damage	Specific for coagulation	Severity& risk of mortality	Recovery	Co‐infections	Ref.
WCC and N	+							Lu et al.^101^
Lymph	+				+	+		Chen et al.^102^
E	+					+		Chen et al.^102^
Plt	+				+	+		Chen et al.^102^
L/N ratio	+				+	+		Assandri et al.^103^
RDW	+	+			+			Henry et al.^23^
MDW							+	Ciaccio et al.^20^
PCT		+					+	Polilli et al.,^104^ Silberbauer^37^
CRP	+				+	+		Assandri et al.^103^
SaO2					+			Assandri et al.^103^
cTnI/cTnT			+		+			Du et al,^10^
NT‐proBNP			+					Han et al.^105^
Hyc		+	+		+			Ponti et al.,^86^ Yang et al,^88^
D‐dimer, FDP and PT	+	+		+	+	+		Han et al.,^106^ Tang et al.,^70^
IL−6 and Ferritin		+			+	+		Velavan et al.^107^
SAA					+			Li et al.^14^
LDH		+				+		Tian et al.^108^
Mb and CK‐MB			+		+			Qin et al.,^59^ Parohan et al.^63^

Note

(+) means that the marker is characterized as the considered property.

Abbreviations: ALT, Alanine transaminase; CK‐MB, creatine kinase‐myocardial band; CRP, C‐reactive protein; E, eosinophil; FDP, fibrin degradation products; Hyc, homocysteine; LDH, lactate dehydrogenase; Lymph, lymphocyte; Mb, myoglobin; N, neutrophil; Plt, platelet; PT, prothrombin time; RDW, red blood cell distribution width; SAA, serum amyloid A; SaO2, oxygen saturation; WCC, white cell count.

**TABLE 2:** CV biomarker modification following the COVID‐19 progression

Biomarkers	Alternation	Timing	Diagnostic consideration
cTnI/cTnT	Increased	_	Critical indicators for two purpose: Identifying a silent myocardial injury, Reliable biomarkers to risk assessment if the patients require ICU care.
D‐Dimer and PT/aPTT	Increased	Day 4	D‐dimer levels can be used as a prognostic marker. Increased levels of d‐Dimer have positive relationship with ICU admission and mortality rate. The prolong PT also was indicated as non‐survival factor.
CK‐MB	Increased	_	CK‐MB is a biomarker of myocardial injury and reperfusion. Raised CK‐MB levels in COVID−19 are correlated with infarct size and a predictor of poor prognosis in ICU‐admitted patients.
BNP/NT‐proBNP	Increased	_	There is conflicted data. Both increased and unchanged levels of BNP/NT‐proBNP were reported in different cohort studies.
Fibrinogen & Plt	Decreased	6–10 days after admission	Platelet count is a simple, afordable, rapid and accessible laboratory parameter to discriminate between non‐severe and severe COVID−19 patients
Hyc	Increased	_	A novel indicator for thromboembolism events and platelet aggregation, used for the severity determination in COVID−19 patients

Abbreviations: BNP/NT‐proBNP, Brain natriuretic peptide/NT‐proBNP; CK‐MB, creatine kinase‐myocardial band; cTn, cardiac troponins; cTnI, cardiac troponin I; cTnT, cardiac troponin T; CV, cardiovascular; Hcy, homocysteine; Plt, platelet count; PT, prothrombin time.

#### 3.2.3 Auto‐antibodies

The appearance of some auto‐antibodies can also precipitate thrombotic events in some patients. A recent case report described three COVID‐19 patients whose anticardiolipin IgA, anti‐β2‐glycoprotein I IgA and IgG antibodies were positive during their evaluations.^74^ To note, these antibodies are commonly detected in the sera of patients with antiphospholipid syndrome and occasionally in those with critical illness or infection.^75^ Indeed, making an accurate diagnosis and differentiating from other causal factors involved in multifocal thrombosis in critically ill patients, such as heparin‐induced thrombocytopenia, consumptive coagulopathy and thrombotic microangiopathy, would be challenging.

### 3.3 Time interval required for retrieving the modified biomarkers after treatment process

**FIGURE 2:**
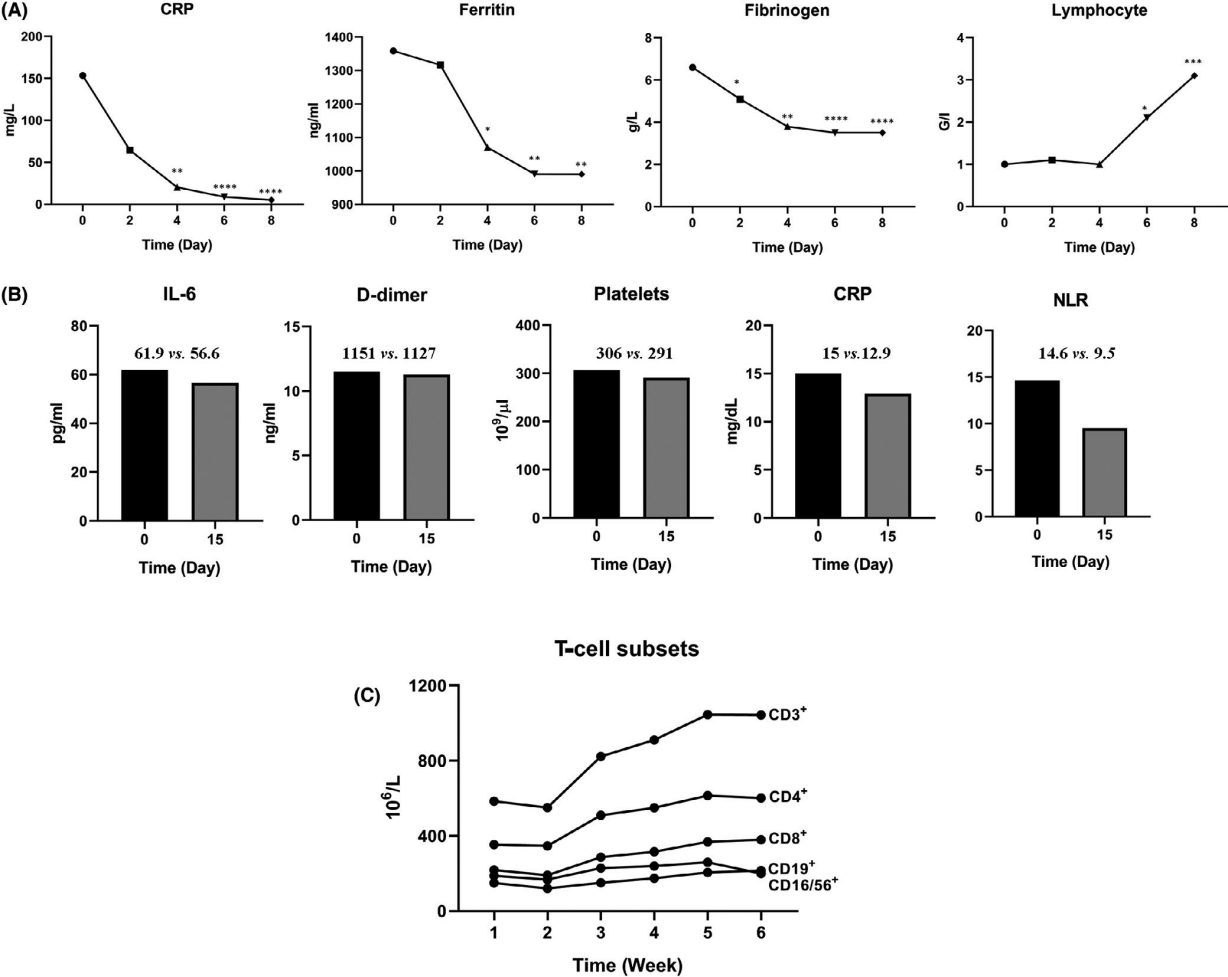
Time interval of biomarkers alternation during the recovery process after treatment. According to the various studies, some biomarkers amounts after recovery in infected patients including CRP (mg/L), Ferritin (ng/ml), Fibrinogen (g/L), lymphocytes (G/L), IL‐6 (pg/ml), d‐dimer (ng/ml), platelet (×10^9^/µl), NLR and T‐cell subsets (×10^6^/L) were reported. (A) The mean biomarkers levels after tocilizumab administration in patients with ARDS (*n* = 40 patients), *p < 0.05; **p < 0.01; ***p < 0.005; ****p < 0.001; [Adapted from the *Clinical and Experimental Rheumatology*; 2020],^76^ (B) median baseline of haematological and inflammatory values in response to sarilumanb (*n* = 15 patients) [Adapted from the *Journal for ImmunoTherapy of Cancer*; 2020],^77^ (C) T‐cell subsets counts in COVID‐19 patients who recovered (*n* = 415 patients) [Adapted from the *International Journal of Infectious Diseases*; 2020]^32^

For better managing the critically severe form of COVID‐19, several anti‐inflammatory medications were applied. In this respect, tocilizumab (TCZ) and sarilumab, as known anti‐IL‐6 monoclonal antibodies, exerted appreciable therapeutic outcomes. In limited literatures, the time interval of some important values was also reported during the treatment process. In Figure 2, using GraphPad software, the trend of indicators fluctuation during the recovery process has been brought. In a study conducted by Conrozier et al., it was reported that the elevated level of CRP dramatically retrieved at days 4 or 5 (−86.7%, *p* < 0.0001) and rebounded to the reference range as early as day 6 in COVID‐19 patients who successfully treated with TCZ for 8 days.^76^ Additionally, in these patients, the abnormal levels of ferritin, fibrinogen and lymph were also returned to the normal range within 6 day (990.7 ng/ml, *p* < 0.01, 3.5 g/L, *p* < 0.0001 and 3.1 G/I, *p* < 0.005 respectively) (Figure 2A). In the case of sarilumab, a single‐centre cohort study also analysed the haematological and inflammatory values changes in patients with COVID‐19. Out of 15 patients, 10 subjects showed a significant improvement corresponded with sarilumab upon 15 days. Following the treatment, the total level of WCC proceeded to decrease between 3 and 15 days and the declined levels of lymph count increased between 4 and 10 days (*p* < 0.01). Despite a downward trend of CRP, IL‐6, NLR and d‐dimer amounts, ferritin could not return to the normal values up to day 6 (Figure 2B).^77^ Based on the study conducted by Deng et al., T‐cell subsets in recovered patients began to decline upon 2 weeks and returned to the reference range within 4–6 weeks (Figure 2C).^32^

### 3.4 Biomarker changes following COVID‐19 therapy

According to the published data, it has been demonstrated that the levels of some dynamic biomarkers could be affected by COVID‐19 therapies. In this regard, administration of corticosteroids, especially low‐to‐moderate dexamethasone doses as a substantial part of therapies for patients with severe form, are being conducted by various ongoing interventional clinical trials (ClinicalTrials.gov: NCT04619693, NCT04707534, NCT04836780, NCT04327401 and NCT04395105). Despite a significant improvement in the clinical manifestations, for example 28‐day survival, ICU length of stay and duration of mechanical ventilation,^78^ there are limited data regarding the possible beneficial effects of corticosteroids on both cardiac and non‐cardiac serum biomarkers during COVID‐19.^79^ However, it has also been reported that corticosteroid therapy in younger patients (≤65 years of age) and females displayed a significant improvement in CRP, IL‐6 and d‐dimer serum levels (*p*≤0.05).^78^ In contrast, a recent clinical study indicated that the levels of plasma IL‐6 in 62.5% of hospitalized patients (>10 pg/ml) were still higher after dexamethasone administration, which is likely related to the co‐express of the glucocorticoid receptor (GR or NR3C1) and IL‐6.^80^ To note, the effect of corticosteroids on soluble IL‐6 receptor upregulation has also been well‐established, previously.^81^ In COVACTA clinical trial, TCZ was used to determine prognostic biomarkers for clinical outcomes among 295 hospitalized patients. According to the results, it has been revealed that the high levels of ferritin, as a predictive value, but not CRP reduction, were directly associated with improved clinical outcomes in TCZ recipients compared with the placebo group at day 28.^82^, ^83^ Moreover, the effect of subcutaneous injection of single dose sarilumab is also being conducted to evaluate possible benefits compared to the standard of care in the early stages of the disease. Even so, data have not been published yet.^84^ In a retrospective case series, the implications of sarilumab administration also showed a rapid dropping in CRP levels corresponded with clinical improvement. In addition, lower levels of IL‐6 and NLR ratio were observed in a large portion of patients.^77^

## 4 NEW EMERGING BIOMARKERS

Besides the commonly used laboratory parameters, some novel biomarkers such as homocysteine (Hcy), Ang II, Ang‐(1–7) and alamandine are highly suggested for further investigation as candidates for the new diagnosis values, particularly in the prognosis of the severe form of COVID‐19 with CVD manifestations. Regarding the cardio‐protective concept of the RAS, Ang (1–7) and alamandine exert the vasodilator effect by increasing nitric oxide release from the endothelium and the decreasing nicotinamide adenine dinucleotide phosphate oxidase‐related superoxide production.^85^ Despite existing limited data, the anti‐inflammatory effect of Ang‐(1–7) and alamandine has been established in both *in vitro* and *in vivo* settings.^85^ Moreover, a recent piece of evidence postulated that the plasma levels of Hcy, as a new predictive value for CV risk, enhance (>the 90th percentile) following both macro‐ and microangiopathies, especially in the coronary and peripheral vasculatures. Moreover, it is thought to be that the increased levels of Hcy are considered as a risk factor for thromboembolism events by affecting the plt activity as a potential biomarker to determine the severity in COVID‐19 patients,^86^ which subsequently can promote cellular death through ferroptosis.^87^, ^88^ Recently, mounting evidence highlighted the therapeutic and diagnostic (theranostic) potential of exosomes, defined as secreted extracellular nano‐sized vesicles serving a substantial role in intercellular cargo transportation (eg lipids, proteins and microRNAs) through biological fluids, as well as drug delivery tools, under various pathophysiological conditions such as endocrinology, CVD, cancer and inflammation.^89^, ^90^ In the case of COVID‐19, it has also been proposed that exosomes either have the potential to involve in COVID‐19 pathogenesis or would be of utmost importance as novel and advanced biomarkers, particularly in personalized diagnostic and therapeutic criteria.^91^, ^92^ Notably, a proteomic profile related to exosomes in the critically and non‐critically ill patients, mainly thought to be related to inflammation, coagulation and immune response, was assessed.^93^ In this regard, it has been reported that tenascin‐C (TNC) and fibrinogen‐β (FGB) were abundantly found in circulating exosomes of COVID‐19 patients.^94^ Differentially expressed exosomal circular RNA (circRNA) and long non‐coding RNAs (lncRNA), involved in immunity and inflammation regulation, can also substantiate a predominant role of exosomes in disease pathogenesis.^95^ In this regard, the therapeutic potential of T‐cell‐derived exosomes is being elegantly evaluated against pneumonia in early‐stage COVID‐19 administered by aerosol inhalation.^96^

## 5 THE EFFECTS OF DEMOGRAPHIC FACTORS ON CV RELATED BIOMARKERS

Given the importance of demographic factors, for example age and gender, in CVD pathophysiology, it should also be considered whether they can affect CV‐related biomarkers in the setting of COVID‐19, although the exact role of sex differences on dynamic biomarkers, particularly cardiac parameters, is needed longitudinal follow‐up studies to gain better insight into the long‐term CV impacts in COVID‐19 patients; however, sex differences are becoming a more imperative issue as accumulating evidence indicates that males seem to be abundantly at higher risk of severe COVID‐19 due to either pre‐existing CVD or incidence of COVID‐19‐related CV injury, which can exaggerate by age.^97^ It is also well‐established that ageing is taken into account as one of the leading host risk factors beyond the cytokine storm, NLRP3/inflammasome, air pollution and hypoxemia to manifest COVID‐19‐induced CV morbidity in older people, which finally results in serious consequences and increased mortality rate.^98^ Regarding the sex differences, Cheng et al. declared that the incidence of myocardial injury among men was higher than women hospitalized due to the COVID‐19 (9.2 vs. 4.9%, *p* = 0.004).^99^ Notably, it can be inferred that affecting ACE2 expression by sex hormones may underlie presumable sex differences in cardiotropic SARS‐CoV‐2 infection.^97^ In line with this claim, a recent study reported that androgen signalling has a regulatory effect on ACE2 expression following the SARS‐CoV‐2 infection in human cardiac cells.^100^ Overall, male patients with COVID‐19 experience poorer clinical outcomes in comparison with age‐matched female patients. Based on logistic regression analysis, two factors of age and chronic kidney disease were directly associated with myocardial injury in male and female patients.^99^

Moreover, WCC, hs‐CRP and d‐dimer were independent risk factors in male patients strongly correlated with hs‐cTnI and BNP changes.^99^ It has also been proposed that the inflammatory biomarkers in males were significantly higher than in females in the course of disease progression, highlighting the stronger immune responses in women and robust inflammatory activation in males. According to the results of a recent clinical study, early and peak levels of CRP IL‐6, PCT and ferritin changed in men remarkably more than women (*p* < 0.05), leading to higher ICU admission and mortality rate in men.

## 6 CONCLUSIONS

Last but not least, CV events, as one of the serious complications during COVID‐19, appeal a close following up in one site and further attempt to raise the public health knowledge on the other side. Considering undeniable both Cv and non‐CV evolving COVID‐19 complications, determination of prognostic and diagnostic criteria is of especial interest in modifying optimized therapeutic protocols and minimizing medical bias, particularly in the management of critically ill patients with pre‐existing or COVID‐19‐induced CVD. To this end, the clinical surveillance accompanied by early assessment and persistent monitoring of cardiac‐specific biomarkers including cTnI/T, NT‐proBNP, Mb and CK‐MB, as well as coagulation indicators such as d‐dimer, thrombocyte count, PT and Hyc, may identify timely cardiac injury and predict COVID‐19 complications after hospitalization. However, publishing the results of ongoing clinical trials would pave the way for better clinical decisions in medical care centres.

## CONFLICT OF INTEREST

The authors have no perceived competing of interest related to this work.

## AUTHOR CONTRIBUTION

**Aysa Rezabakhsh:** Conceptualization (lead); Investigation (lead); Supervision (equal); Writing – original draft (lead); Writing – review & editing (equal). **Seyyed‐Reza Sadat‐Ebrahimi:** Writing – review & editing (equal). **Alireza Ala:** Writing – review & editing (equal). **Seyed Mohammad Nabavi:** Writing – review & editing (equal). **Maciej Banach:** Investigation (lead). **Samad Ghaffari:** Conceptualization (lead); Supervision (lead).
